# Variance aftereffect within and between sensory modalities for visual and auditory domains

**DOI:** 10.3758/s13414-023-02705-5

**Published:** 2023-04-26

**Authors:** Sachiyo Ueda, Reiko Yakushijin, Akira Ishiguchi

**Affiliations:** 1 Department of Computer Science and Engineering, Toyohashi University of Technology, 1-1 Hibarigaoka, Tempaku-cho, Toyohashi, Aichi 441-8580 Japan; 2 Department of Psychology, Aoyamagakuin University, Tokyo, Japan; 3 Faculty of Core Research, Ochanomizu University, Tokyo, Japan

**Keywords:** Temporal variability, Ensemble perception, Adaptation aftereffect, Cross modality, Size, Pitch

## Abstract

We can grasp various features of the outside world using summary statistics efficiently. Among these statistics, variance is an index of information homogeneity or reliability. Previous research has shown that visual variance information in the context of spatial integration is encoded directly as a unique feature, and currently perceived variance can be distorted by that of the preceding stimuli. In this study, we focused on variance perception in temporal integration. We investigated whether any variance aftereffects occurred in visual size and auditory pitch. Furthermore, to examine the mechanism of cross-modal variance perception, we also investigated whether variance aftereffects occur between different modalities. Four experimental conditions (a combination of sensory modalities of adaptor and test: visual-to-visual, visual-to-auditory, auditory-to-auditory, and auditory-to-visual) were conducted. Participants observed a sequence of visual or auditory stimuli perturbed in size or pitch with certain variance and performed a variance classification task before and after the variance adaptation phase. We found that in visual size, within modality adaptation to small or large variance, resulted in a variance aftereffect, indicating that variance judgments are biased in the direction away from that of the adapting stimulus. In auditory pitch, within modality adaptation to small variance caused variance aftereffect. For cross-modal combinations, adaptation to small variance in visual size resulted in variance aftereffect. However, the effect was weak, and variance aftereffect did not occur in other conditions. These findings indicate that the variance information of sequentially presented stimuli is encoded independently in visual and auditory domains.

The world that we perceive is rich in variability. For example, during a conversation, people’s facial expressions and voices change in a short period of time. In cities, cars with various shapes, colors, and sizes run off intersections with different engine noises. The stock price of the economic market changes constantly. Perceiving the variability of things in the outside world is important for predicting the subsequent event and taking an appropriate action. In the field of behavioral economics, variance in outcomes or uncertainty is considered risk, which is known to play a major role in human decision-making bias (Kahneman & Tversky, 1979). In the real world, however, variance is rarely explicitly given. Thus, we have to use our perceptual systems to extract the implicit magnitude of variability from a vast amount of sensory input. Decision-making is then performed based on the perceived magnitude of variability. Therefore, it is important to understand how we perceive the variability of things or events.

It has been discussed in the studies of “ensemble perception” whether and how the human perceptual system can perceive variability (or variance) of stimuli. Ensemble perception is the ability of the human perception system to instantly and efficiently perceive summary statistical information (such as mean and variance) of the entire stimulus, beyond the processing of individual stimuli (for a review, Whitney & Yamanashi Leib, 2018). So far, there has been a great deal of interest in average perception. There have been studies on averaging of various stimulus features, from low-level dimensions such as orientation (Parkes et al., 2001; Robitaille & Harris, 2011), size (Ariely, 2001; Attarha et al., 2014; Chong & Treisman, 2003, 2005), luminance (Bauer, 2009), length (Weiss & Anderson, 1969), speed (e.g., Emmanouil & Treisman, 2008), direction of motion (Watamaniuk & Dunchon, 1992), to high-level dimensions such as facial expressions (Haberman et al., 2009; Haberman & Whitney, 2009), gender (Haberman & Whitney, 2007), and liveliness (Yamanashi Leib et al., 2016). By perceiving the average, it is possible to reduce the effect of noise derived from individual stimuli and to grasp the central tendency of the stimuli set. (Alvarez, 2011).

In contrast, variance is an index representing the degree of homogeneity of the stimuli. Although less attention has been paid to variance perception compared to average perception, in recent years increasing evidence has been reported on people also being able to perceive the variance in various visual elements—from lower-level stimuli such as orientation (Morgan et al., 2008; Solomon, 2010), luminance (Tong et al., 2015), motion (Suárez-Pinilla et al., 2018), size (Solomon et al., 2011; Ueda et al., 2018), and color (Bronfman et al., 2014; Maule & Franklin, 2020; Michael et al., 2014; Ward et al., 2016) to higher-level stimuli such as facial expressions (Haberman et al., 2015) and race (Phillips et al., 2018). The duration time of stimulus presentation in the previous studies was approximately 150–1,000 ms, indicating that observers can perceive variance in a short time. It was also suggested that color variability was extracted very rapidly and preattentively (Bronfman et al., 2014; Michael et al., 2014) and without awareness of individual features (Ward et al., 2016). Based on an ideal observer analysis using a computational process model, Solomon et al. (2011) argued that variance perception is more accurate than average perception and that late noise, which has been theorized to impact average perception, does not affect variance perception.

Furthermore, while it has been pointed out that mean and variance representations interact with each other (Jeong & Chong, 2020; Michael et al., 2014; Tong et al., 2015), some studies support the possibility that variance perception involves an encoding mechanism independent of mean perception (Khvostov & Utochkin, 2019; Maule & Franklin, 2020; Norman et al., 2015; Yang et al., 2018). For example, Norman et al. (2015) showed that prolonged exposure to high or low orientation variance shifts the perceived magnitude of orientation variance of the subsequent texture stimuli in a direction away from that of the adapting stimulus, even when the mean orientation changes between adaptation and test stimuli (i.e., negative variance aftereffect). Similarly, variance aftereffect could occur in color when the mean hue presented on each slide varies at random during adaptation (Maule & Franklin, 2020). These findings suggest “direct encoding” of variance, independent of the central tendency (Maule & Franklin, 2020; Norman et al., 2015) and highlight the existence of a neural substrate specifically attuned to variance.

It should be noted that these studies on variance perception have focused on spatial integration in situations where all stimuli are presented simultaneously. However, in everyday life, people sometimes encounter multiple stimuli, such as people or animals, one after another over time (i.e., sequential presentation). Even if the target groups are spatially gathered in a certain area, it may not be possible to see it at once, and objects in different locations may be processed sequentially by eye movement or exploratory actions (i.e., sequential sampling). In addition, in a situation where the environment changes constantly, knowing the temporal variance of stimuli leads to prediction of the next state and preparation to adapt. Therefore, it is necessary to integrate the stimuli over time and determine the characteristics of the entire stimulus. Average perception is possible in a temporal context (Albrecht et al., 2012; Albrecht & Scholl, 2010; Haberman et al., 2009; Hubert-Wallander & Boyton, 2015; Yamanashi Leib et al., 2016), and observers can perceive averages in temporal or sequential presentations as accurately as in spatial or simultaneous presentation (Chong & Treisman, 2005). Regarding variance perception, few studies have focused on the variance of stimuli presented in a sequence (Payzan-LeNestour et al., 2016; Ueda et al., 2015, 2018). In particular, Payzan-LeNestour et al. (2016) asked participants to rate the magnitude of perceived volatility of movement trajectories or numerosity that changed over time on a 5-point scale and demonstrated the variance adaptation effect. This suggests that there is a specific mechanism for perceiving variance in temporally or sequentially presented stimuli as well as in spatially or simultaneously presented stimuli (Maule & Franklin, 2020; Norman et al., 2015).

In addition, statistical summaries of stimuli presented over time may be highly important and essential in the auditory modality. Auditory system discriminates “sound texture” in auditory scene using time-average statistics (McDermott et al., 2013). Listeners can estimate average frequency of a set of logarithmically spaced pure tones presented in a temporal sequence, even when they are poor at identifying and determining the positions of the individual tones due to a brief presentation time (Piazza et al., 2013). The average auditory pitch can be grasped at least as accurately as a visual stimulus (Albrecht et al., 2012; Piazza et al., 2013). However, studies on variance perception have been limited to the visual domain.

Thus, in this study, we focused on variance perception in the temporal context and investigated whether any variance aftereffects occur in each of the visual and auditory domains. For visual stimuli, we employed a circle with changing size, and for auditory stimuli, we changed the pitch of pure tones. Both are relatively low-level features; we can determine whether variance perceptions in the visual and auditory domains have any common characteristics by examining the possibility of each variance aftereffect using the same psychophysical procedure.

Moreover, we also tested whether the adaptation aftereffect of variance perception occurred across different modalities. Variance extraction has unique characteristics that differ from the mean, which is a highly common statistic across different stimuli attributes and sensory modalities (Maule & Franklin, 2020; Ueda et al., 2014, 2018). For example, when there are different-sized oranges and matchsticks lined up in different orientations, it is nonsensical to compare the average size with that of the orientation. However, it is possible to judge to a certain extent which set (i.e., orange vs. matchstick) has greater variation. This unique characteristic of variance as a statistical index raises questions about variance perception: Is there a common mechanism that does not depend on stimulus attributes in variance perception? Indeed, in the visual system, the domain-general mechanism of variance perception has been reported. Maule and Franklin (2020) showed that adaptation to hue variability changed the judgment of orientation variability, that is, the variance aftereffect occurred across different visual properties. Similarly, Payzan-LeNestour et al. (2016) showed that the variance aftereffect could occur in the temporal variability of numerosity even when adaptation and test stimuli were given as quite different representations. These examples of variance adaptation aftereffects between different stimulus features suggest a domain-general mechanism of variance perception in the visual system.

Given the characteristics of variance as a statistical indicator that is common to different sensory modalities, it is possible to extend the domain-general variance perception mechanism to cross-modality. Ueda et al. (2018) examined the effect of audition on visual variance perception using auditory stimuli with a pitch corresponding to the visual size. It was shown that synchronized presentation of auditory and visual stimuli that have the same variance improves the precision of perceived variance in size, when compared with the condition of only visual stimuli presentation. This suggests the possibility that although participants were instructed to ignore the auditory stimuli, the pitch variance of the auditory tones might have been automatically sampled and pooled with the sizes of the visual stimuli, which may have influenced the perceived size variance. In line with this study, we investigated herein whether the domain-general variance perception system exists across different sensory modalities by using the variance adaptation aftereffect paradigm.

In light of the preceding discussion, we propose two hypotheses: The first hypothesis is that there are mechanisms for direct encoding of the variance of sequentially presented stimuli in both visual and auditory domains. In the experiment, the mean value was varied on each trial; therefore, if a negative variance aftereffect occurred after prolonged exposure to either small or large variance, it would indicate that the variance was encoded independently of the mean (i.e., “direct encoding” of variance). Maule and Franklin (2020) and Norman et al. (2015) examined the variability of spatially distributed visual stimuli and found variance aftereffects. In contrast, our study used auditory and visual stimuli, presented these stimuli as distributed over time, and asked the participants to classify variance of the stimuli as relatively small or large (the method of single stimuli; McKee et al., 1986). The second hypothesis posits the existence of a sensory-domain-general mechanism for variance perception. If cross-modal adaptation aftereffects are observed in variance perception, it would yield novel and significant insight into the existence of variance perception mechanisms common across different sensory modalities. In Experiment 1, we tested the variance aftereffect under the conditions of “within visual size” (unimodal aftereffect: VV) and “visual size to auditory pitch” (cross-modal aftereffect: VA). In Experiment 2, the conditions of “within auditory pitch” (unimodal aftereffect: AA) and “auditory pitch to visual size” (cross-modal aftereffect: AV) were tested.

## Experiment 1

### Methods

#### Participants

The participants were 12 paid volunteers (two females and 10 males, ages 20–24 years). The sample size was based on previous visual variance adaptation studies (Maule & Franklin, 2020; Norman et al., 2015). It corresponded to an effect size *d* = 0.8 (one-sample *t* test, one-tailed), alpha = 0.05, power = 0.8 using the G*Power 3.1 (Faul et al., 2007, 2009). A total of thirteen participants participated in Experiment 1, as one was excluded from the analysis due to poor fit of psychometric function. They were graduate students and undergraduate students at the Toyohashi University of Technology. All participants provided written informed consent prior to participating in the experiment. All participants had normal or corrected-to-normal vision and were naïve to the purpose of the study. Experiments 1 and 2 were approved by the Ethical Committee for Human-Subject Research at Toyohashi University of Technology, and all experiments were performed in accordance with the committee’s guidelines and regulations. 

#### Apparatus

We generated visual and auditory stimuli using MATLAB with the Psychtoolbox extension (Brainard, 1997; Kleiner et al., 2007; Pelli, 1997) on a MacBook Pro computer. Visual stimuli were displayed on a 17-inch CRT monitor with a resolution of 1,152 × 864 pixels (refresh rate of 75 Hz). For each observer, auditory stimuli were presented at the same moderate volume via headphones (SONY MDR-CD900ST). Participants sat in a dimly lit room and, using a chin rest, observed the monitor from a distance of approximately 57 cm. For each experimental trial, they pressed a key on an Apple keyboard to provide a response.

#### Stimuli

In each test trial, eight white disks on the visual condition or pure tones in the auditory condition were presented sequentially. The diameters of each disk and the frequencies of each tone were randomly chosen from the lognormal distribution, lnN (lnD, σ^2^) and lnN (lnF, σ^2^), respectively. Standard deviation (σ) were determined for each condition. The baseline frequency and diameter were changed for each sequence, and the *baseline* diameter D was randomly selected between 1.0° and 1.2°, and the *baseline* frequency F was randomly selected between 200 Hz and 300 Hz. Each element was shown for 150 ms, with a 100-ms blank interval between successive elements. In the adaptation phase, a sequence consisting of eight white discs, similar to the test stimuli, was presented 100 times with a 300 ms interval between the sequences. The standard deviation (σ) of diameter was set to 0.05 for small variance adaptation conditions, and set to 0.6 for large variance adaptation conditions. In the test phase, there were six conditions for magnitudes of variance: for visual size, standard deviations (σ) were set to 0.2, 0.25, 0.3, 0.35, 0.4, 0.45, and for auditory pitch, standard deviations (σ) were set to 0.175, 0.275, 0.375, 0.475, 0.575, and 0.675, respectively. As regards the variance for these visual and auditory test stimuli, the authors tried various values in advance and selected those values that fit the psychometric function. Pure tones were heard using headphones. The disks were positioned on an outline of an invisible circle with a radius of 1°, which was located at the center of a gray background screen. The luminance of the gray background was 1.22 cd/m^2^ (CIE xy chromaticity coordinates, *x* = 0.266, *y* = 0.433), and the luminance of the white disk was 91.4 cd/m^2^ (CIE xy chromaticity coordinates, *x* = 0.256, *y* = 0.337).

#### Procedure

All participants experienced the following four conditions: two adaptation conditions (either small or large variance) each in VV (visual size adaptor and visual size test) and VA (visual size adaptor and auditory pitch test) conditions. To avoid participant fatigue, each condition was divided into two sessions. The eight sessions were conducted in pseudorandom order on separate days. Each session lasted approximately 30 min.

Before each session, the participants underwent 24 training trials (four trials for each variance magnitude condition) to get used to the variance classification task to be performed in the test phase. They were asked to classify the magnitude of variance of the six levels of test stimuli using a two-alternative forced choice between relatively small or relatively large (a method of single stimuli). Although an explicit reference standard was not provided, participants were given feedback on whether their answers were correct. Feedback was also given during the preadaptation test phase to establish the implicit standard for variance classification. If variance adaptation distorts variance perception in the postadaptation test, then classification performance of perceived variance against the memory-based implicit standard should change.

Each session consisted of a preadaptation test phase, an adaptation phase, and a postadaptation test phase. In each trial of the preadaptation test phase, a fixation point was presented in the center of the screen for 500 ms. Next, a sequence of visual or auditory stimuli was presented, in which the elements’ size or pitch was perturbed with one of the six magnitudes of variance. Participants were required to classify the variance of the stimuli as relatively small or large by pressing *1* on the keyboard if the variance was perceived as relatively small and *3* if it was perceived as relatively large. The correct answers were to respond with “large” for the three largest conditions and to respond with “‘small” for the three smallest conditions out of the total six conditions of variance magnitudes. The feedback was presented for 500 ms before the next trial started. After 120 preadaptation test trials (20 trials for each variance magnitude condition), a short break was taken before initiating the adaptation phase. Before starting the adaptation phase participants were reminded to make sure to keep looking at the stimuli during visual stimuli adaptation. In the adaptation phase, a sequence consisting of eight visual stimuli with a certain variance (σ = 0.6 for large adaptation condition; σ = 0.05 for small adaptation condition), similar to that in the test stimulus, was presented in 100 trials. Each sequence had the same variance, but the averages differed between the sequences. The interval between the sequences was 300 ms. The posttest phase started immediately after the adaptation phase. In the postadaptation test phase, participants performed a variance classification task, similar to that in the preadaptation test; however, in this phase the participants were not given feedback. In addition, the blocks of six adaptation trials (top-up adaptation) were interleaved with those of six test trials to maintain the effect of variance adaptation (i.e., six adaptation trials and six test trials were alternatively presented). Thus, there were, in total, 120 postadaptation test trials and 120 adaptation trials across 20 blocks in the posttest phase.

### Results and discussion

**Fig. 1:**
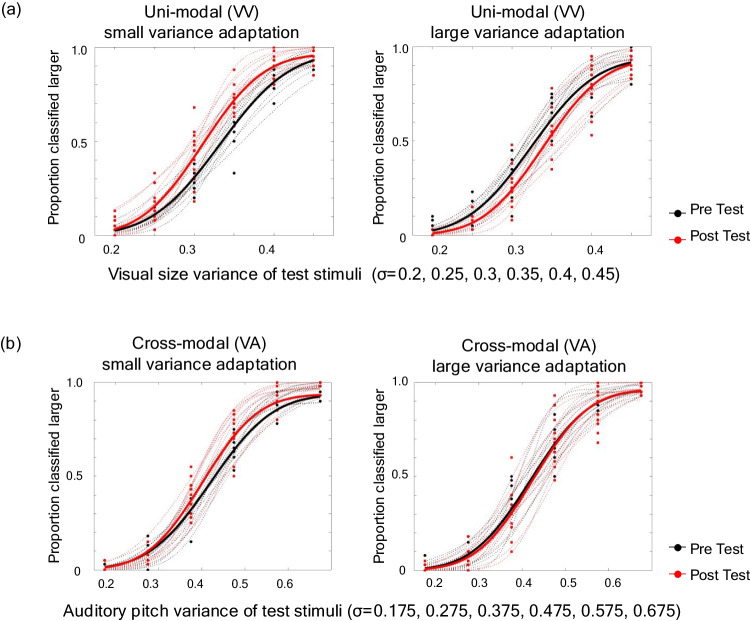
Experiment 1 results: the data of **(a)** unimodal (VV: visual-to-visual) and **(b)** cross-modal (VA: visual-to-auditory) conditions. Left panels show the small variance adaptation, and right panels show the large variance adaptation. Proportion of classified larger was plotted for each variance magnitude in pretest (red) and posttest (black), and the PSE was calculated by fitting a cumulative normal function and finding the 50% point for each participant. Dotted lines show each participant’s data, and bold lines showed the average across all participants. (Color figure online)

**Fig. 2:**
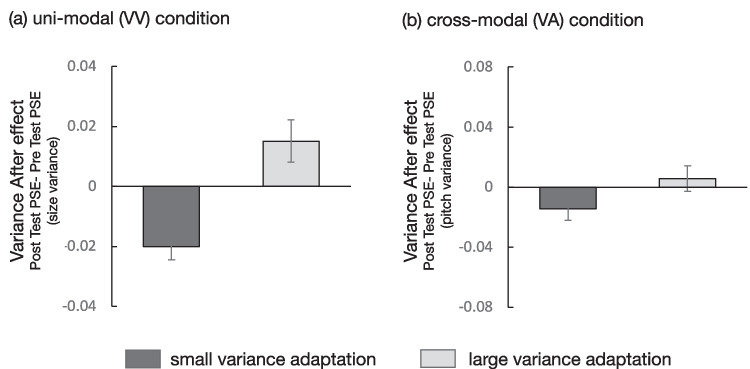
Experiment 1 results: PSE shift (posttest PSE – pretest PSE) in the **(a)** unimodal (VV) condition and (**b**) cross-modal (VA) condition. One-sample *t* test (one-tailed) showed that PSE shifted in the expected direction (i.e., variance aftereffect) in the VV small variance adaptation (*p* < .001), VV large adaptation (*p* = .028), and VA small adaptation (*p* = .039) conditions. Error bars show ±1 standard error of the mean (*SEM*)

A boundary for variance classification, that is, the point of subjective equality (PSE) at which participants were equally likely to classify the variance of stimuli as small or large, was calculated for the pre-and postadaptation test trials by fitting a cumulative normal function. The calculation was conducted for each participant using the Palamedes toolbox (Prins & Kingdom, 2009) in MATLAB. One participant was excluded from the analysis because of poor fit of the psychometric function. Figure 1 shows the proportion classified “larger” for each variance magnitude of test stimuli. Four graphs were drawn for each experimental condition (VV small variance adaptation, VV large variance adaptation, VA small variance adaptation, and VA large variance adaptation), with the pretest (black line) and posttest (red line) plotted for each participant (dashed line) and the average of all participants (thick line). If aftereffect occurs, the PSE shifts toward the adaptation stimulus. To verify whether PSE shifted in the expected direction, the magnitude of PSE shift (postadaptation PSE − preadaptation PSE) was calculated for each participant and averaged for all 12 participants (Fig. 2). A one-sample *t* test (one-tailed) was then conducted to test if the PSE shift was different from zero for each condition. We found that the PSE shifted in the expected direction under the VV small adaptation condition (t=−4.412, *p* < .001, *d* = −1.274), VV large adaptation condition (*t* = 2.112, *p* = .028, *d* = 0.620), and VA small adaptation condition (*t* = −1.948, *p* = 0.039, *d* = 0.562). Under the VA large condition, PSE shifted in the expected direction but was not significant relative to zero (*t* = 0.6728, *p* = .258, *d* = 0.194).

These results indicate that when the adaptor and test stimuli were presented in the same modality, perceived visual size variance was significantly larger after prolonged exposure to small variance adaptors; the opposite occurred following adaptation to large variance adaptors. This is consistent with previous studies that showed a variance adaptation aftereffect of orientation (Norman et al., 2015) and color (Maule & Franklin, 2020) on simultaneous presentation of ensemble stimuli. The current study showed that variance aftereffect occurred even when stimuli with varying visual size were presented one by one in time. Notably, however, the small variance adaptation condition showed a very large effect size, whereas the effect of the large variance condition was relatively small.

The other interest in this study was to examine whether the variance aftereffect occurs between different modalities (i.e., the cross-modal aftereffect). In this experiment, we tested whether prolonged exposure to the visual adaptor distorted the variance perception of auditory pitch and found that the cross-modal aftereffects occur only under the small variance adaptation condition. This result suggests that there may be variance aftereffect across modalities, but they are likely to be weak. Given that the variance aftereffect under the small variance adaptation condition was strong in the unimodal condition, it is plausible that the cross-modality condition, which would typically be less prone to produce aftereffect, might have yielded aftereffects only under the small variance adaptation condition.

## Experiment 2

Experiment 1 showed that adaptation to visual size variance distorted visual variance perception (i.e., negative variance aftereffect), but the effect on variance perception of auditory pitch was partial and weak. In Experiment 2, we tested whether adaptation to auditory pitch variability caused an aftereffect in the variance perception of auditory pitch and visual size.

### Methods

#### Participants

The participants were 12 paid volunteers (all male, ages 20–24 years). Two participants participated in Experiment 1. They were graduate students and undergraduate students at the Toyohashi University of Technology. All participants provided written informed consent prior to participating in the experiment. All participants had normal or corrected-to-normal vision and were naïve to the purpose of the study.

#### Apparatus

For Experiment 2, the same apparatuses as those used in Experiment 1 were applied.

#### Stimuli

The stimuli were the same as those in Experiment 1, except for the following. In the adaptation trials, eight pure tone sequences were presented 100 times. The STANDARD DEVIATION (σ) of frequency was set to 0.05 for the small variance adaptation condition and to 0.8 for the large variance adaptation condition.

#### Procedure

The procedure was almost the same as that in Experiment 1, but the experimental conditions were changed. All participants experienced the following four conditions: two adaptation conditions (either small or large variance) each in the AA (auditory pitch adaptor and auditory pitch test) and AV (auditory pitch adaptor and visual size test) conditions. In the adaptation phase, participants observed a sequence of auditory stimuli perturbed in pitch with a certain (large or small) variance.

### Results and discussion

**Fig. 3:**
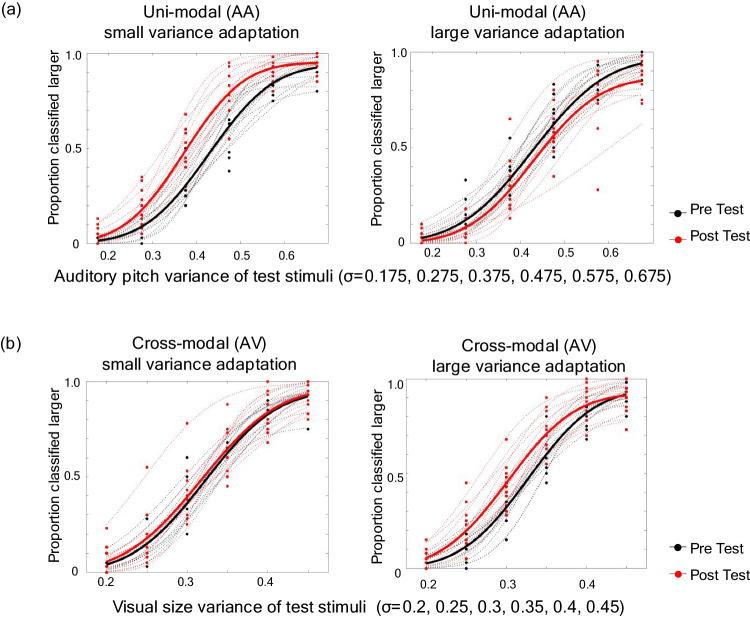
Experiment 2 results: **(a)** unimodal (AA: auditory-to- auditory) and **(b)** cross-modal (VA: auditory-to-visual) conditions. Left panels show the small variance adaptation, and right panels show the large variance adaptation. Proportion of classified larger was plotted for each variance magnitude in pretest (red) and posttest (black), and the PSE was calculated by fitting a cumulative normal function and finding the 50% point for each participant. Dotted lines show each participant data, and bold lines showed the average across all participants. (Color figure online)

**Fig. 4:**
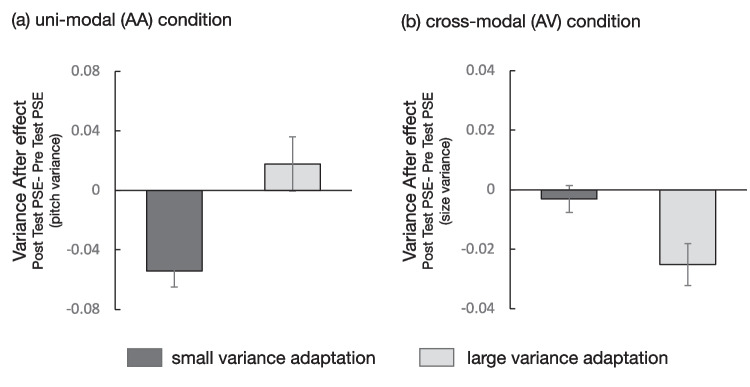
Experiment 2 results: PSE shift (posttest PSE – pretest PSE) in **(a)** unimodal (A A) condition and **(b)** cross-modal (AV) condition. One-sample *t* test (one-tailed) showed that PSE shifted in the expected direction (i.e., variance aftereffect) in the AA small variance adaptation condition (*p* < .001). Error bars show ±1 standard error of the mean (*SEM*)

The point of subjective equality (PSE) was calculated for the pre-and postadaptation test trials, similar to Experiment 1. Figure 3 shows the proportion classified “larger” for each variance magnitude of test stimuli. Four graphs were drawn for each experimental condition (AA small variance adaptation, AA large variance adaptation, AV small variance adaptation, and AV large variance adaptation), with the pretest (black line) and posttest (red line) plotted for each participant (dashed line) and the average of all participants (thick line). The magnitude of PSE shift (postadaptation PSE − preadaptation PSE) was calculated for each participant and averaged for all 12 participants (Fig. 4). A one-sample *t* test (one-tailed), conducted to test whether the PSE shift was different from zero for each condition, showed that PSE shifted in the expected direction only in the case of the AA small adaptation condition (*t*=−4.980, *p* < .001, *d* = –1.438). Under the AA large adaptation condition (*t* = 0.968, *p* = .177, *d* = 0.279) and the AV small adaptation condition (*t* = –0.382, *p* = .355, *d* = –0.11), PSE shifted in the expected direction but was not significantly different from zero. Under the AV large condition, PSE shifted in the direction opposite to that expected, (*t* = −3.695, *p* = .998, *d* = −1.067).

As in the visual domain, we found the variance aftereffect to be in the expected direction in the AA small adaptation condition. Perceived auditory pitch variance was significantly larger after prolonged exposure to small variance adaptors when the adaptor and test stimuli were presented in the same modality. However, unlike in the visual domain, variance aftereffect did not occur in the unimodal large variance adaptation condition (AA large variance condition). This may indicate that auditory pitch variance perception has different characteristics than visual size variance perception. Alternatively, considering that the variance aftereffect was relatively weak even under the VV large variance condition, it is possible that when stimuli are presented over time, the variance aftereffect is less likely to occur when the stimulus is adapted to a large variance. This point will be discussed later. Under the cross-modal condition, variance aftereffect did not occur in the expected direction.

## General discussion

The results of our study demonstrate that adaptation to variance in multiple visual or auditory stimuli presented sequentially can distort the variance perception of the subsequent test stimuli within the same modality. That is, adaptation to sequences of visual stimuli with a small variance caused the variance of the subsequent visual stimulus to be perceived as more variable, and adaptation to large variance in visual stimuli caused the variance of the subsequent visual stimulus to be perceived as less variable. In the auditory domain, only small variance adaption caused the variance aftereffect. Whereas variance aftereffect for orientation (Norman et al., 2015) and color (Maule & Franklin, 2020) have been shown in the context of spatial integration (i.e., ensemble items presented simultaneously), this study found that the variance aftereffect can also occur in the context of temporal integration. As the mean of size and pitch varies in every trial, the aftereffect is the evidence that the variability of the stimuli is encoded independently of the central tendency (“direct” encoding of variance, as discussed in Maule & Franklin, 2020; Norman et al., 2015). If the aftereffects were based on adaptation to individual stimuli, stimuli with varying mean would not have produced an aftereffect. Moreover, to the best of our knowledge, this study is the first to demonstrate the auditory pitch variance aftereffect. Although average perception of auditory pitch has been reported (Albrecht et al., 2012; Piazza et al., 2013), no studies have investigated its variance perception. In the variance judgment task of our experiment, the response “relatively large” increased monotonically with respect to the levels of the variance presented, showing that the participants were able to perceive, to some extent, the different magnitudes of auditory pitch variance. This study suggests that the variance of auditory pitch is encoded independently of the mean, similar to that of visual stimuli.

However, cross-modal variance aftereffect was observed only under the VA small variance adaptation condition, in which adaptation to visual stimuli with small variance caused auditory stimuli to be perceived as more variable. This means that, unlike the perception of motion (Berger & Ehrsson, 2016; Kitagawa & Ichihara, 2002; Konkle et al., 2009) or numerosity (Arrighi et al., 2014) that show cross-modal negative aftereffects, a common neural substrate in variance perception across different sensory modalities seems unlikely; at least variance perception mechanisms are segregated between sensory modalities at lower encoding levels.

There are several points to note in the results. First, a negative variance aftereffect within the same modality occurred after prolonged exposure to small variance adaptors in both visual and auditory domains. Large variance adaptation in visual domain resulted in a relatively weak variance aftereffect (effect size was small) but not in auditory domain. This suggests that adaption to large variance is unlikely to have occurred in our experiment. This, as it were, unidirectional aftereffect (i.e., adaptation only caused the stimulus set to appear highly varied) contrasts with the findings of bidirectional variance aftereffect (which showed the aftereffect in both large and small variance adaptation) reported in the context of spatial integration of orientation (Norman et al., 2015) and color (Maule & Franklin, 2020). The difference in our results may be due to the presentation of the stimulus (i.e., temporal or sequential presentation vs. spatial or simultaneous presentation). The bidirectional variance aftereffect observed in the previous studies can be explained by the multichannel account: variance is processed by multiple channels, each tuned to a specific range of variance magnitude, or at least variance representation is determined by two channels—one tuned to a small variance and the other tuned to a large variance (Morgan et al., 2008; Norman et al., 2015). However, the unidirectional aftereffect observed in this study does not match the multichannel account. Rather, a mechanism sensitive to the difference in small variance might explain the unidirectional aftereffect within the same modality in our study. That is, multiple channels that finely tune the difference in small variance, so to speak the homogeneity detector, might work in variance perception of sequentially presented stimuli set. Another possible reason is that the stimulus for the larger variance adaptation contained extreme values, and the prominence of the extreme value may have reduced adaptation to the stimulus by not attending the whole stimulus. Additionally, the possibility of sampling error or sampling leakage due to sequential presentation should be considered. The variance perception mechanism in the context of temporal integration needs to be investigated further using stimuli of other properties such as orientation to examine whether the characteristics of the variance aftereffect can be generalized.

Second, adaptation to large variance of auditory pitch biased the perceived variance of visual size toward “large.” This is a change in the direction opposite to that expected and does not indicate a mechanism of variance perception common to the visual–auditory domain. It is, however, currently difficult to explain why such a positive bias occurred only in the AV large condition.

Third, in this study, participants were asked to judge the relative magnitude of variability of the stimuli in comparison to the implicit standard. Our experiment was conducted based on the assumption that the implicit standard remained constant following adaptation, but the perceived variability of posttest stimuli was affected by adaptation. Although we believe that the consistent trends observed across each condition in this study suggest the effect at the perceptual level, it remains possible that the adaptation of variability may have altered the implicit standard. For example, if the implicit standard shifted towards a smaller variability in the VV small condition, then the perceived variability of the posttest stimuli would likely be perceived as relatively large. If adaptation changed the implicit standard stored in memory, it would imply an adaptation effect at a higher cognitive processing level than the perceptual level. Though some studies have suggested that the perception of variability in orientation occurs at a relatively early cortical stage (Norman et al., 2015) and is an automatic, attention-independent process (Durant et al., 2017), the extent to which variance aftereffect reflects lower-level perceptive process or higher-order cognitive process remains to be established in the case of sequentially presented ensemble stimuli. Accordingly, more research is needed to explore the mechanism of variance aftereffect.

Finally, one may argue that the distortion of variance perception in this study was derived from decisional bias. We cannot completely deny that the clear difference in variance magnitude in the adaptation phase facilitated decisional bias among the participants. For example, when a small variance adaptor is repeatedly presented, it is possible that the participants imagined the intention of the experimenter and consciously judged the magnitude of the variance to be large. However, this is unlikely to apply to our study. The participants in our study were naïve to this study’s purpose. If it were a decisional bias, it would not have yielded consistent results as the direction of the bias would have varied among individuals. Additionally, even if there was a bias to make decisions in the opposite direction of the variance magnitude of the adaptive stimulus, there should have been a systematic bias corresponding to the magnitude of the adaptive stimulus under all conditions. Variance aftereffects, however, were observed only under the unimodal condition. Therefore, we believe that the results of this study cannot solely be explained by decisional bias.

It is a limitation of this study that we did not implement any task to ensure that participants were attending to the visual stimuli during the adaptation phase. However, participants were instructed to ensure that they were paying attention to the visual stimuli during the adaptation phase, and after the experiment, the experimenter verified that these instructions were followed.

In conclusion, in both the auditory and visual domains, negative variance aftereffects occur within the sensory modality, especially in the adaptation to small variance. In contrast, the aftereffects for cross-modality were limited. These findings indicate that the variance information is encoded independently for each sensory modality. In the visual domain, variance aftereffects occur across different attributes and presentation formats, suggesting a domain-general variance perception mechanism (Maule & Franklin, 2020; Payzan-LeNestour et al., 2016). The current study suggests a direct encoding mechanism for the variance of stimuli presented across time in both the auditory and visual domains, but abstract variance representations may be specific to sensory modality.

## Data Availability

All raw data files are available at Mendeley Data DOI (10.17632/j95kbcfp75.1). It will be made public on a permanent repository if the manuscript is accepted for publication. None of the experiments was preregistered.

## Code availability

The software application and custom code are not available.
